# A Genome-Wide Association Study of First-Episode Psychosis: A Genetic Exploration in an Italian Cohort

**DOI:** 10.3390/genes16040439

**Published:** 2025-04-07

**Authors:** Mirko Treccani, Lucia Maggioni, Claudia Di Giovanni, Laura Veschetti, Doriana Cristofalo, Cristina Patuzzo, Antonio Lasalvia, Branko Ristic, Roushan Kumar, The PICOS-Veneto Group, Mirella Ruggeri, Chiara Bonetto, Giovanni Malerba, Sarah Tosato

**Affiliations:** 1 GM Lab, Department of Surgical Sciences, Dentistry, Gynaecology and Paediatrics, University of Verona, 37134 Verona, Italy; mirko.treccani@univr.it (M.T.); giovanni.malerba@univr.it (G.M.); 2 Department of Neuroscience, Biomedicine and Movement Sciences, Section of Psychiatry, University of Verona, 37134 Verona, Italy; lucia.maggioni@univr.it (L.M.); doriana.cristofalo@univr.it (D.C.); antonio.lasalvia@univr.it (A.L.); branko.ristic@univr.it (B.R.); roushan.kumar@univr.it (R.K.); mirella.ruggeri@univr.it (M.R.); chiara.bonetto@univr.it (C.B.); 3 Department of Diagnostic and Public Health, University of Verona, 37134 Verona, Italy; claudia.digiovanni@univr.it; 4 Infections and Cystic Fibrosis Unit, Division of Immunology, Transplantation and Infectious Diseases, IRCCS San Raffaele Scientific Institute, 20123 Milano, Italy; veschetti.laura@hsr.it; 5 Vita-Salute San Raffaele University, 20123 Milano, Italy; 6 Department of Neuroscience, Biomedicine and Movement Sciences, University of Verona, 37134 Verona, Italy; cristina.patuzzo@univr.it

**Keywords:** first-episode psychosis, schizophrenia, genome-wide association study, neurodevelopment disorder

## Abstract

Background: Psychosis, particularly schizophrenia (SZ), is influenced by genetic and environmental factors. The neurodevelopmental hypothesis suggests that genetic factors affect neuronal circuit connectivity during perinatal periods, hence causing the onset of the diseases. In this study, we performed a genome-wide association study (GWAS) in a sample of the first episode of psychosis (FEP). Methods: A sample of 147 individuals diagnosed with non-affective psychosis and 102 controls were recruited and assessed. After venous blood and DNA extraction, the samples were genotyped. Genetic data underwent quality controls, genotype imputation, and a case-control genome-wide association study (GWAS). After the GWAS, results were investigated using an in silico functional mapping and annotation approach. Results: Our GWAS showed the association of 27 variants across 13 chromosomes at genome-wide significance (*p* < 1 × 10^−7^) and a total of 1976 candidate variants across 188 genes at suggestive significance (*p* < 1 × 10^−5^), mostly mapping in non-coding or intergenic regions. Gene-based tests reported the association of the SUFU (*p* = 4.8 × 10^−7^) and NCAN (*p* = 1.6 × 10^−5^) genes. Gene-sets enrichment analyses showed associations in the early stages of life, spanning from 12 to 24 post-conception weeks (*p* < 1.4 × 10^−3^) and in the late prenatal period (*p* = 1.4 × 10^−3^), in favor of the neurodevelopmental hypothesis. Moreover, several matches with the GWAS Catalog reported associations with strictly related traits, such as SZ, as well as with autism spectrum disorder, which shares some genetic overlap, and risk factors, such as neuroticism and alcohol dependence. Conclusions: The resulting genetic associations and the consequent functional analysis displayed common genetic liability between the non-affective psychosis, related traits, and risk factors. In sum, our investigation provided novel hints supporting the neurodevelopmental hypothesis in SZ and—in general—in non-affective psychoses.

## 1. Introduction

Psychosis is characterized by delusions, hallucinations, and incoherent behavior or speech. It can be divided into two main diagnostic categories: affective psychosis, such as major depression with psychotic features and bipolar disorder, and non-affective psychosis, which includes schizophrenia (SZ) and schizophrenia spectrum disorder (SSD) [1]. The incidence of psychosis in Italy is 18.1 per 100,000 people per year. Regarding the diagnostic subcategories, the incidence rates are 5.6 per 100,000 per year for schizophrenia, 14.3 per 100,000 per year for schizophrenia spectrum disorder, and 3.8 per 100,000 per year for affective psychoses [2].

Psychosis is a complex and multifactorial group of disorders, which is influenced by both genetic and environmental factors [3], and its underlying etiopathogenesis still remains poorly understood.

One of the explanations that guided research on SZ throughout this decade is the neurodevelopmental hypothesis. The idea was discussed in 1986 by D. R. Weinberger who thought that the perinatal period was crucial for the combined effect of genetic and environmental hostile aspects on dysfunctional brain development [4].

Among environmental factors, obstetric complications (OCs) are a well-known risk factor for SZ [5] and poor clinical outcomes [6]. Moreover, an interaction between SZ polygenic risk scores and OCs was demonstrated in increasing the risk of SZ [7], even if this result was not confirmed [8]. Alongside this, gene expression studies have shown that genes involved in SZ are more expressed during fetal development compared to postnatal [9,10]. Epigenetic studies demonstrate that SZ risk loci are enriched for fetal DNA methylation marks, indicating a developmental window of vulnerability [11,12]. Furthermore, interactions between genetic risk and OCs suggest that prenatal adversity amplifies the SZ risk [13]. The placenta emerges as a critical mediator, with SZ risk genes dynamically expressed during complicated pregnancies, reflecting cellular stress and immune responses [7].

Various Genome-Wide Association Studies (GWASs) reported several single nucleotide polymorphisms (SNPs) associated with SZ or related traits. To date, the largest GWAS in SZ, conducted by the Schizophrenia Working Group of the Psychiatric Genomics Consortium (PGC), found 342 linkage-disequilibrium-independent SNPs (*p* < 5 × 10^−8^) in 287 genetic risk loci [14]. Among these, relevant risk-associated SNPs are located in genes, such as ZNF804A (rs1344706), which belong to the zinc finger protein family of transcription factors and has a role in neural connectivity [15], through MIR137HG (rs11165917, rs4274102), which controls the expression of other genes by binding to the 3′ untranslated region of their transcripts and is involved in synaptic genesis and plasticity [16], and ATP2A2 (rs4766428), which is responsible in calcium signaling [14]. The SNPs are generally responsible for small effects on the risk of SZ [17], and since most of them are typically located in non-protein-coding regions, they are likely involved in gene expression regulation that, in turn, may affect brain development and function [13].

Currently, the GWASs have only focused on SZ diagnosis but not on the broad non-affective psychosis, with the exception of two studies where the disease status was based on self-reported psychotic experiences [18,19]. However, to our knowledge, a genome-wide analysis investigating people with a former diagnosis of non-affective psychosis at their first episode of psychosis (FEP) is lacking.

In this study, we performed a GWAS for non-affective psychosis in a cohort of first-episode psychosis (FEP individuals and matched controls to extend previous results related not only to SZ but also to non-affective psychosis). Our hypothesis is that the risk variants for non-affective psychosis are on genes expressed more during the perinatal period compared to later stages of neurodevelopment, such as childhood and adolescence.

## 2. Materials and Methods

### 2.1. Participants

Patients were recruited within the broader framework of the Psychosis Incident Cohort Outcome Study (PICOS), which is a multicenter, longitudinal, prospective study on FEP conducted in the Veneto Region, Italy [20]. Patients at their first psychotic episode were recruited from public Community Mental Health Centers (CMHCs) participating in the project. The primary goal of PICOS is to provide a detailed characterization of the clinical presentation and outcomes of FEP patients and to evaluate the relative influence of various risk factors—environmental, social, biological, genetic, and neuroanatomical—in order to develop a predictive model for psychosis outcomes. Detailed information on the study design and recruitment process has been reported elsewhere [6,20,21]. Patients were included in the study if they (i) were aged between 15 and 54 years; (ii) were residents in the Veneto Region (Italy); (iii) experienced, for the first time, at least one of the following positive symptoms: hallucinations, delusions, qualitative speech disorder, qualitative psychomotor disorder, and bizarre or grossly inappropriate behavior; or at least two of the following negative symptoms: loss of interest, initiative, and drive, social withdrawal, episodic severe excitement, purposeless destructiveness, overwhelming fear, or marked self-neglect; and (iv) made initial contact with CMHCs for the aforementioned symptoms. Patients were excluded according to the following criteria: (i) having a history of antipsychotic treatment exceeding three months, (ii) having mental disorders attributable to a general medical condition, or (iii) having moderate to severe intellectual disability.

Nine months after enrollment, the diagnosis was confirmed using the Item Group Checklist (IGC) of the SCAN [22], and only patients who received an ICD-10 diagnosis of psychosis were included in the study. For the purpose of this research, we analyzed only the “non-affective psychosis” (F1x.4, F1x.5; F1x.7; F20; F21, F22, F23, F25, F29) sample from the PICOS cohort.

A control group was recruited through an advertisement posted at the University Hospital of Verona (Italy) [23]. Healthy subjects were matched by age and sex to the patients and were all negative for physical or psychiatric disorders, traumatic brain injury, and intellectual disability. The absence of psychiatric disorders was confirmed using the Mini International Neuropsychiatric Interview (M.I.N.I. Plus) [24] and the Mini International Neuropsychiatric Interview (SCID-II) [25].

The study was approved by the Ethics Committee of Azienda Ospedaliera of Verona, Italy, with protocol code 1103, and all participants provided written informed consent. For all participants, socio-demographic characteristics were collected.

### 2.2. DNA Extraction and Genotyping

From each participant, venous blood samples (15 mL) were obtained using EDTA-containing tubes. DNA was then isolated from peripheral blood leukocytes with a commercial kit (ABgene, Blenheim Road, Epson, Surrey, UK). Genotyping was performed at the Institute of Psychological Medicine and Clinical Neurology, Cardiff University, UK, utilizing custom Illumina HumanCoreExome-24 BeadChip arrays, which included probes for 570,038 genetic variants (Illumina, San Diego, CA, USA). Genotype calling was performed using the Illumina GenomeStudio 2.0 software package.

### 2.3. Genomic Analysis

After genotyping, genetic data underwent quality controls using PLINK 1.90 version b7.7 [26], considering the following exclusion criteria: genotype missingness > 0.02, sample missingness > 0.02, minor allele frequency (MAF) < 0.005, and Hardy–Weinberg equilibrium test *p*-value < 1 × 10^−10^ [27]. Sex information concordance between genetic and clinical data was checked, and individuals having discordant sexual information were discarded. Related individuals were assessed using the kinship coefficients: related individuals up to 2nd degree were discarded. Only individuals of European descent were retained. Genotype imputation [28] was performed on the TOPMed Imputation Server [29] using the default settings and the TOPMed r3 reference panel. Imputed variants were filtered according to their imputation quality (R2) and minor allele frequency: variants with a MAF > 0.01 and R2 > 0.3 or with a MAF ≤ 0.01 and R2 > 0.7.

Genome-wide and suggestive significance thresholds were arbitrarily set to *p* < 1 × 10^−7^ and *p* < 1 × 10^−5^, respectively.

Suggestive and genome-wide significant findings were searched for already reported associations within the GWAS catalog, querying the database version e0_r2022-11-29 [30]. Traits were tested for enrichment using hypergeometric tests and Bonferroni corrections.

### 2.4. In Silico Functional Analyses

Moreover, to understand the functional and biological relevance of the genetic findings with non-affective psychosis or related traits, in silico functional analyses were carried out, comprising variant annotations, gene sets enrichment analysis, and search for already reported associations. The analysis was performed using the SNP2GENE and GENE2FUNC modules from FUMAGWAS version 1.5.2 [31], using ANNOVAR version 2017-07-17 [30] for variant annotations; MAGMA version 1.08 for gene sets enrichment analysis [32] on the Genotype-Tissue Expression (GTEx) project version 8 [33] and BrainSpan v10 [34]; and eQTL and chromatin interaction analyses on publicly available data from GTEx v8, PsychENCODE version DER-08a [35], BrainSpan v10 [34], and BRAINEAC v1 [36].

## 3. Results

### 3.1. The PICOS Participants

A total of 249 individuals (147 diagnosed with non-affective psychosis and 102 controls, matched per sex and age) were genotyped. Among the 147 patients recruited, 62 (42.2%) were female with a mean age of onset of 29 years old (SD 9 years), 42 (28.6%) were diagnosed with SZ, and 105 (71.4%) with other non-affective psychosis. Further socio-demographic and clinical characteristics of the sample are illustrated in Table 1. Regarding genetics, 432,089 genetic markers were imputed. After imputation and data cleaning, a total of 13,365,611 genetic markers, either typed or imputed, underwent genetic association analysis.

### 3.2. Genome-Wide Association Analysis of Non-Affective Psychosis

**Figure 1:**
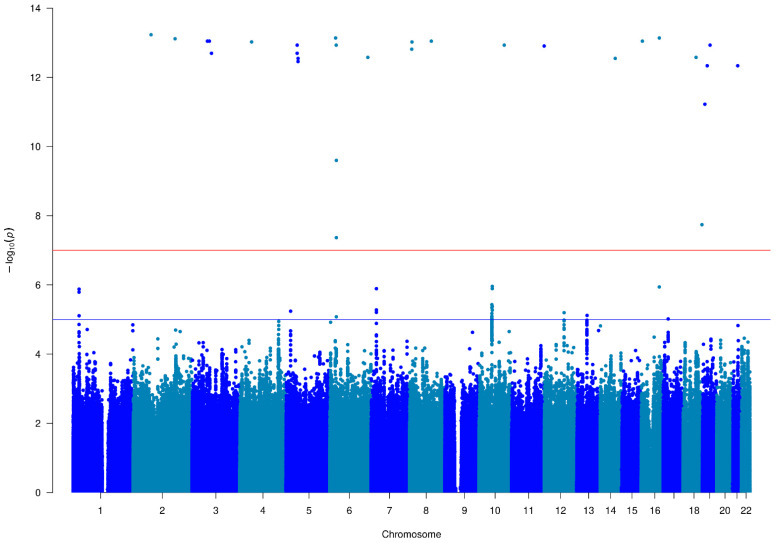
GWAS results in non-affective psychosis. The figure shows a Manhattan plot reporting the genetic associations with the non-affective psychosis. Genome-wide (*p*-value < 1 × 10^−7^) and suggestive (*p*-value < 1 × 10^−5^) significance thresholds are reported with the red and blue lines, respectively. Even and odd chromosomes are reported in light and dark blue, respectively. The list of significant and suggestive associations is reported in Supplementary Table S2.

Among the over 13 million genetic markers investigated (typed: 432,089; imputed: 12,933,522), a total of 27 variants across 13 chromosomes turned out to be associated with non-affective psychosis at a genome-wide significance level (*p* < 1 × 10^−7^), as shown in Table 2. Additionally, 55 variants were significantly associated at a suggestive level (*p* < 1 × 10^−5^), as reported in Figure 1.

The GWAS Catalog was queried to identify previously reported associations. Most variants matched traits related to schizophrenia (rs72897474, rs9665626, rs7206782, rs7253952, rs7591150) and waist-to-hip ratio adjusted for BMI (rs7206782, rs13270070, rs9657509). Additionally, related terms, such as alcohol dependence (rs3131513), autism spectrum disorder (rs9393484), bipolar disorder (MTAG) (rs9665626), educational attainment (rs7606483), general cognitive ability (rs7606483), and neuroticism (rs13270070), were identified. Several variants in MFHAS1 (rs2271342, rs12677543, rs12682352, rs2409091, rs9644776, rs60315134, rs59046059, rs11784052, rs35039922, rs57312668, rs4841051, rs1039916, rs3789849, rs7820146, rs3925830, rs332037, rs332039, rs332040) and SLC25A12 (rs59844139) were linked to autism spectrum disorder. The rs142968358 SNP in SPTLC1P2 was also correlated with this condition. Associations with intellectual disability include rs78294462 in TCF4, rs376456 and rs1009136 in MAU2, and rs7200247 in GLG1. Additional variants in SUFU (rs17114641, rs9665626, rs729025, rs3824756, rs7086898, rs11191356, rs11191359, rs4146429) were also implicated. Finally, general cognitive ability was linked to rs11126396 in RNU6-111P and RPSAP28.

Moreover, a manual literature search harbored additional information regarding variants associated with epilepsy, such as rs12713794 in the TPRKB gene and multiple SNPs (rs2979172, rs2921064, rs2979181, rs13270194, rs7823056) in the PRAG1 gene and with epileptic encephalopathy, such as rs59844139 SNP in SLC25A12 and rs10883761 in TRIM8.

**Figure 2:**
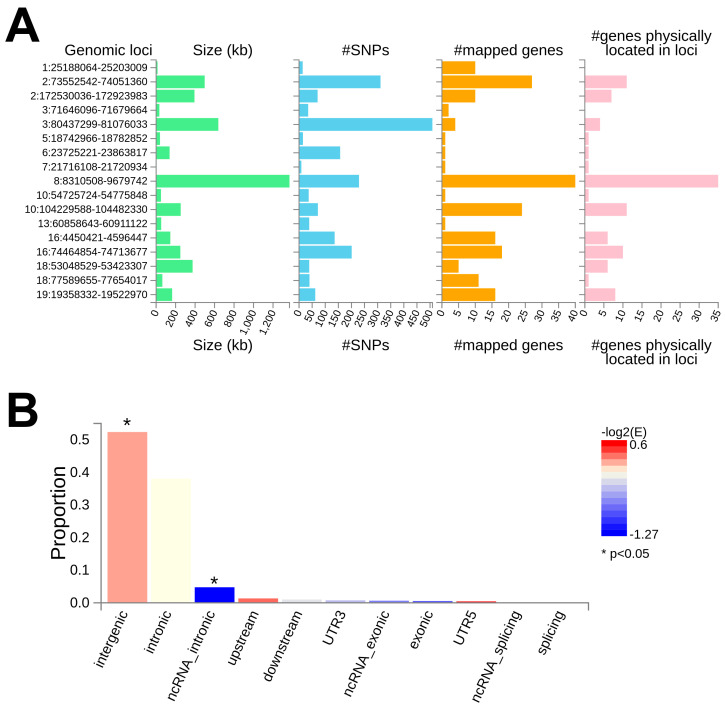
Summary of the genetic associations. The figure reports a summary of the genetic loci associated with non-affective psychosis, providing information on (**A**) the identified genomic risk loci and (**B**) the functional consequences of the associated SNPs. For each genomic risk locus (**A**) the information regarding the number of genes resulting from the functional analysis (#mapped genes) and located in the identified genomic region (#genes physically located in loci) is reported.

The analysis of the GWAS summary statistics with FUMAGWAS’s SNP2GENE module detected 13 independent genomic regions located on 9 chromosomes and comprising 1976 candidate SNPs across 188 genes, as reported in Supplementary Table S1. Intergenic variants (proportion = 54.0%) were enriched (enrichment = 1.20, *p*-value = 6.77 × 10^−16^), and ncRNA intronic variants (proportion = 4.76%) were less present than expected by chance (enrichment = 0.413, *p*-value = 9.59 × 10^−26^); a comprehensive overview is provided in Figure 2.

**Figure 3:**
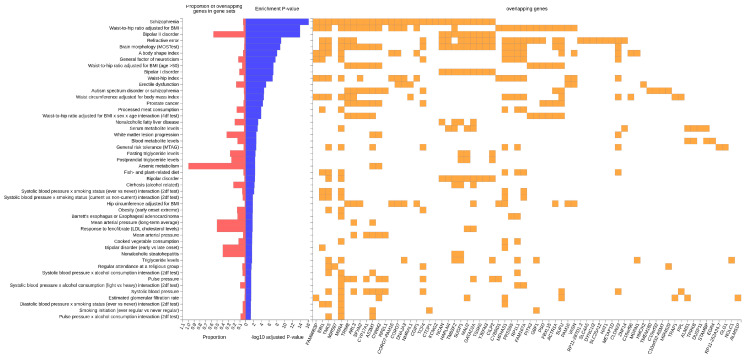
Enriched traits in the GWAS catalog associated with the non-affective psychosis genes. For each trait, the common genes between the GWAS results and those already reported in the GWAS catalog are displayed and identified as overlapping genes.

Furthermore, when searching for associated genes, a total of 48 traits from the GWAS catalog were identified. The most significant trait was “Schizophrenia” (29/659 genes, adjusted *p*-value *p* = 9.41 × 10^−17^), followed by “Waist-to-hip ratio adjusted for BMI” (28/747 genes, adjusted *p*-value *p* = 1.28 × 10^−14^), and “Bipolar II disorder” (9/16 genes, adjusted *p*-value *p* = 1.28 × 10^−14^), as reported in Table 3 and Figure 3.

### 3.3. In Silico Functional Analysis

In silico functional analyses were performed to address possible biological insights from the signals of GWAS.

**Figure 4:**
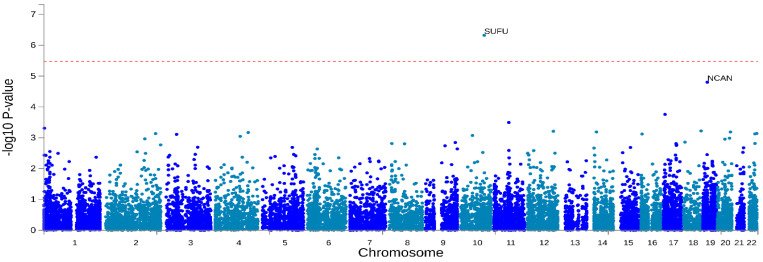
Gene-based association analysis. The figure shows a Manhattan plot reporting the associations of genes with non-affective psychosis. The significance threshold is reported with the dashed red line (*p* = 0.05/14,946 = 3.34 × 10^−6^). Even and odd chromosomes are reported in light and dark blue, respectively.

Investigated variants (13,365,611 typed and imputed markers) were mapped to 15,248 protein-coding genes. A genes-based test showed a single significant association in the SUFU Negative Regulator Of Hedgehog Signaling (SUFU, *p*-value = 4.77 × 10^−7^) with non-affective psychosis when applying the severe Bonferroni correction for multiple testing (nominal *p*-value < 3.34 × 10^−6^). When applying a less restrictive threshold (nominal *p*-value < 10^−4^), a second gene showed a trend of association: Neurocan (NCAN, *p*-value = 1.58 × 10^−5^), as shown in Figure 4.

Gene set enrichment analysis was performed on 169,67 terms from the Molecular Signature Database and the “REACTOME_UREA_CYCLE” set resulted as significantly enriched (n genes = 6; β = 1.956; standard error = 0.3031; adjusted *p*-value = 9.563 × 10^−7^), as reported in Table 4.

**Figure 5:**
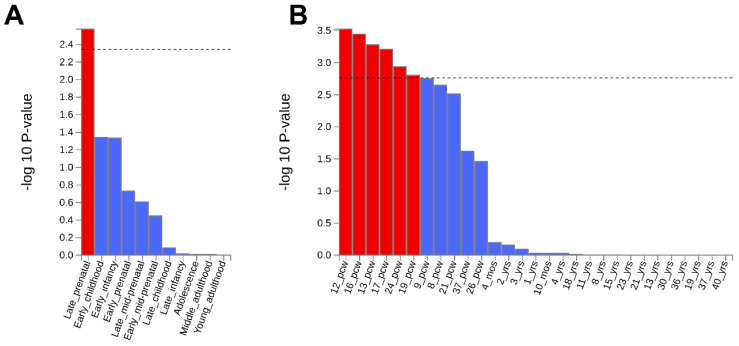
MAGMA expression analysis on BrainSpan. The figure reports the expression analysis performed on the (**A**) 11 general developmental stages and (**B**) 29 different ages of brain samples from BrainSpan. Significant data sets ((**A**): *p* < 4.1 × 10^−3^; (**B**): *p* < 1.5 × 10^−3^) are reported in red.

An analysis investigating whether the set of genes harboring the SNPs associated with non-affective psychosis were expressed in specific tissues or developmental stages was carried out on the GTEx v8 (comprising 54 specific and 30 general tissue types) and on the BrainSpan (comprising 29 different ages and 11 general developmental stages of brain samples) datasets. Results highlighted an association with the BrainSpan “Late prenatal” gene set (nominal *p*-value = 1.4 × 10^−3^) and with six post-conception weeks (pcw) gene sets, spanning from 12 pcw (*p* = 6.4 × 10^−4^) to 24 pcw (1.4 × 10^−3^), as reported in Figure 5.

## 4. Discussion

In this study, we presented the first GWAS, to our knowledge, on non-affective psychosis in FEP individuals. We identified different genomic associations comprising variants located in genes (such as SUFU and NCAN) already known to be associated with disorders (such as SZ) and traits directly related to non-affective psychosis, as well as non-coding variants, concordant with previous findings [13,17,37,38]. Together with GWAS analysis, we performed a detailed bioinformatic analysis of individual GWAS results, examining the association of biological features—such as genes, gene sets expressed across tissues and developmental stages, and pathways—with various traits to gain a deeper understanding of this diagnosed condition.

Our findings suggested hints for the association of two genes already reported to be associated with SZ, such as SUFU [39,40] and NCAN [41,42,43]. The SUFU gene encodes a protein that regulates the Hedgehog (Hh) signaling pathway, essential for development, tissue repair, and cell growth. As a negative regulator, it drives the correct development of the brain, heart, and limb formation [44]. The NCAN gene encodes neurocan, a proteoglycan essential for nervous system development and extracellular matrix regulation in the brain and spinal cord [41]. Altered NCAN levels or mutations have been associated with SZ, autism spectrum disorder (ASD), and bipolar disorder [42,43].

Moreover, our GWAS suggested not only the association of non-coding variants in known genes but mostly the association of variants in intergenic regions. Rather than representing causative pathogenic variants, these findings might suggest that the real causes of these disorders might reside in the alteration of the regulation of gene expression. Our in silico functional analysis proposed some interactions between non-coding and intergenic variants and the genes physically located in proximity to these variants, suggesting pathways and gene sets that might be further investigated to better understand this condition and validated with in vitro approaches.

Noteworthily, we observed that several genes significantly associated with non-affective psychosis belonged to the BrainSpan “Late prenatal” gene set together with several “post-conception weeks” gene sets (ranging from 12 to 24 pcw), corroborating the neurodevelopmental hypothesis for this disorder. Indeed, numerous studies have demonstrated that the second trimester and the perinatal period are critical for the development of SZ, as this is when synaptogenesis begins and continues into childhood [45]. Additionally, the early stages of an individual’s life, particularly during the fetal period, are marked by a larger expression of genes associated with SZ [9,46], which might have a role in poorer fetal brain development. Importantly, the absence of a significant association with late childhood and adolescence further strengthens the relevance of our findings, since it confirms that the perinatal period is crucial not only for SZ, as widely shown in the literature [9,10,13,47,48], but also for non-affective psychosis.

Interestingly, the gene-sets enrichment analysis showed significance for the urea cycle pathway. Previous literature has shown that disruptions in the urea cycle can produce psychotic symptoms, such as hallucinations [37], due to hyperammonaemia, which alters the neurotransmitter systems [49]. This overlap can sometimes result in misdiagnosis or delayed diagnosis [50,51]. The urea cycle impairment might be in part due to nitric oxide metabolism disruption, which is found abundantly in SZ [52] and is known to be relevant for several neural aspects, such as the formation of synapses, cell migration, and cognitive abilities [53]. Although a recent study could not establish an association between psychotic onset and increased urea cycle activity [54], the observed pathway enrichment may suggest a potential mechanistic connection between urea cycle dysfunction and psychotic symptoms in FEP patients.

Moreover, our findings from the GWAS Catalog analysis revealed an enrichment of traits linked to neurodevelopmental discrepancies, including ASD, educational attainment, and cognitive ability.

Regarding cognitive ability, it is well-established that individuals already at their first episode of psychosis often exhibit cognitive dysfunction [55], which may be related to excessive synaptic pruning, particularly in adolescence, and the loss of cerebral plasticity [56]. Dysfunctional myelination and abnormalities in oligodendrocytes further exacerbate macroconnectivity impairments in SZ, suggesting that both structural and functional changes in brain development play a role in the disorder [37]. These associations were observed in the Transcription Factor 4 (TCF4) gene [57], and in the Gogli Glycoprotein 1 (GLG1) gene [58,59]. Educational attainment is typically negatively associated with SZ; however, this relationship varies by subtype, with some subtypes showing a positive correlation [60]. Genes such as SLC25A12 and SPTLC1P2 are implicated in ASD, which is a developmental disorder that affects the social communication and behavior of an individual [61]. Variants in the SLC25A12 gene—a glutamate and aspartate exchanger—have been associated with neurodevelopment disorders, including ASD and epileptic encephalopathy [62,63]. In contrast to SZ, ASD is a neurodevelopmental disorder that typically manifests in early childhood, concordantly to what is observed in BrainSpan. The shared genetic risk between these two disorders is unsurprising, as both are characterized by abnormal brain activity, further supporting the neurodevelopmental hypothesis of SZ [64]. Neuroticism is one of the personality traits in the Big Five model and is characterized by a tendency to experience negative emotions. Higher levels of neuroticism are frequently observed in individuals with SZ, and several psychiatric conditions are associated with this trait [65]. Emerging GWAS research has identified shared genetic loci linked to both SZ and neuroticism, suggesting that personality traits may also play a role in shaping SZ’s clinical expression [66]. Alcohol misuse is notably more prevalent in individuals with SZ compared to those without the disorder. This addiction complicates SZ’s clinical presentation, often leading to poor treatment adherence and increased clinical complications [67,68].

Additionally, some genes (TPRKB, PRAG1, SLC25A12, and TRIM8) have been identified as associated with epilepsy, a neurological disorder characterized by recurrent seizures, that shares similar genetic vulnerability with SZ [69]. Seizures could be related to underlying neurological dysfunction in the brain, which might also contribute to the psychotic symptoms of SZ [70,71].

This supports the idea of a common underlying genetic background among neurodevelopmental disorders, in which illnesses such as ASD, SZ, intellectual disability, Attention Deficit Hyperactivity Disorder (ADHD), and bipolar disorder might share common pathological mechanisms [72].

This study has some limitations that need to be acknowledged. First, the sample size is relatively small compared to other GWASs, which may limit the statistical power to detect significant associations. This prevented the possibility of developing a cumulative model of association based on a polygenic risk score in this population at the present time. Also, possible unassessed genetic comorbidities could play a role in the observed associations, and future studies may benefit from systematically evaluating these factors. Furthermore, since the study population is Italian, the generalizability of these findings to more genetically diverse populations may be limited. Nonetheless, this is the first GWAS investigating Italian individuals diagnosed with FEP. Our findings advance the understanding of the pathogenic process underlying non-affective psychosis and highlight the need for further large-scale and comprehensive studies to replicate and expand upon our results.

## 5. Conclusions

In sum, this study presents a genomic investigation focusing on a cohort of FEP patients providing evidence supporting the neurodevelopmental hypothesis for non-affective psychosis. Our GWAS predominantly showed associations of intergenic and non-coding variants, suggesting that the causes of non-affective psychoses might reside more in regulation in gene expression rather than in causative genetic variants. Moreover, it suggested the contribution of two genes, SUFU and NCAN. Additionally, the significant association with the prenatal period reinforces the idea of alterations in gene expression that might occur in the very early stage of life, which supports the neurodevelopmental hypothesis. Finally, the multiple traits related to non-affective psychoses resulting from the GWAS Catalog proposed a common genetic background liability with other diseases, with the involvement of obesity and brain development. Overall, our genetic findings displayed a common liability between the non-affective diagnosis, several known risk factors, and related traits, offering additional evidence- on this complex condition in favor of altered expressional changes during development.

## Figures and Tables

**Table 1:** Characteristics of FEP patients. The table shows the socio-demographic and clinical characteristics of the individuals diagnosed with non-affective psychosis in the PICOS cohort and of healthy controls.

	Non-Affective Psychosis N = 147, N (%)	Healthy Controls N = 102, N (%)
Sex		
Female	62 (42.2%)	57 (55.9%)
Male	85 (57.8%)	45 (44.1%)
Age, years (SD)	-	33.89 ± 10.99
At onset, years (SD)	29.68 ± 9.04	-
Educational level	10 missing	
Primary school	5 (3.6%)	13 (12.7%)
Higher school	132 (96.4%)	89 (87.3%)
Living condition	10 missing	1 missing
Alone	13 (9.5%)	22 (21.8%)
With partner and/or children	27 (19.7%)	38 (37.6%)
With parents	-	33 (32.7%)
With other relatives	92 (67.2%)	1 (1%)
With other people	5 (3.6%)	7 (6.9%)
Working status	12 missing	2 missing
Employed	55 (40.7%)	71 (71%)
Unemployed	46 (34.1%)	10 (10%)
Student/Retired/Other condition	30 (25.2%)	19 (19%)
Marital status	16 missing	
Single	101 (77.1%)	54 (52.9%)
Married	21 (16.0%)	40 (39.2%)
Separated/Divorced	9 (6.9%)	8 (7.9%)
Diagnosis		
Schizophrenia	42 (28.6%)	-
Other non-affective psychosis	105 (71.4%)	-

**Table 2:** Genome-wide significantly associated variants. This table reports the most significant variants (*p* < 1 × 10^−7^) associated with non-affective psychosis; variants are sorted for genomic position. Chrom = chromosome; bp = base pair; RSID = variant identifier according to dbSNP; Gene = annotated nearest gene; Distance = distance in base pair to the nearest reported genes.

Chrom	Position (bp)	RSID	Effect Allele	*p*-Value	Gene	Distance (bp)
2	73,735,674	rs7606483	A	5.9 × 10^−14^	ALMS1	0
2	172,897,761	rs7591150	G	7.7 × 10^−14^	METAP1D	0
3	63,842,629	rs832190	A	8.9 × 10^−14^	THOC7	0
3	71,661,862	rs830650	A	8.9 × 10^−14^	RP11-154H23.3	25,302
3	80,670,963	rs72897474	A	2.0 × 10^−13^	RP11-47P18.1	139,230
4	48,471,170	rs7670045	G	9.4 × 10^−14^		
5	45,882,593	rs9763350	A	2.0 × 10^−13^		
5	46,121,882	rs8185209	G	1.2 × 10^−13^		
5	49,478,029	rs8188217	A	3.5 × 10^−13^		
5	49,674,996	rs74865196	A	2.8 × 10^−13^		
6	23,798,867	rs9393484	A	7.3 × 10^−14^	SPTLC1P2	58,059
6	26,211,146	rs9358912	A	1.1 × 10^−13^		
6	26,958,528	rs9467936	G	2.5 × 10^−10^		
6	157,099,430		A	2.6 × 10^−13^		
8	8691,622	rs13270070	T	1.5 × 10^−13^	MFHAS1	0
8	9620,359	rs9657509	A	9.4 × 10^−14^	TNKS	0
8	89,588,626	rs7819570	A	8.9 × 10^−14^		
10	104,300,638	rs9665626	G	1.2 × 10^−13^	SUFU	0
11	133,959,472	rs12802468	G	1.2 × 10^−13^	JAM3	0
14	81,735,565	rs8007595	A	2.8 × 10^−13^	STON2	0
16	4,515,145	rs7206782	A	8.9 × 10^−14^	CDIP1	0
16	74,649,232	rs8047523	A	7.2 × 10^−14^	HSPE1P7	0
18	53,301,359	rs78294462	A	2.6 × 10^−13^	TCF4	0
19	9,764,421	rs200076265	A	5.9 × 10^−12^	ZNF562	0
19	19,358,332	rs7253952	A	4.6 × 10^−13^	NCAN	0
19	31,045,360	rs8102611	A	1.1 × 10^−13^	ZNF536	0
21	33,175,364	rs62221589	A	4.6 × 10^−13^		

**Table 3:** GWAS Traits associated with the non-affective psychosis variants. This table presents the traits listed in the GWAS Catalog that have been identified as associated with the variant linked to non-affective psychosis. The full list of findings is reported in Supplementary Table S3. RSID = variant identifier according to dbSNP; PMID = publication identifier in PubMed; *p*-value = GWAS catalog *p*-value.

Trait	RSID	Genes	*p*-Value	PMID
Schizophrenia	rs7591150	METAP1D	7 × 10^−7^	35396580
	rs72897474	Intergenic	8 × 10^−8^	26198764
	rs9665626	SUFU	5 × 10^−9^	26198764
	rs9665626	ARL3	3 × 10^−9^	27922604
	rs7206782	CORO7-PAM16	6 × 10^−10^	31740837
	rs7206782	DNAJA3	3 × 10^−7^	26198764
	rs7206782	DNAJA3	9 × 10^−10^	35396580
	rs7253952	MAU2	4 × 10^−8^	30285260
	rs9665626	TRIM8, ARL3	4 × 10^−10^	33479212
Waist circumference adjusted for body mass index	rs13270070	PRAG1, RN7SL178P	9 × 10^−18^	34021172, 30239722
	rs9657509	CLDN23, MFHAS1	3 × 10^−8^	34021172
	rs7206782	TNKS	1 × 10^−11^	34021172
Alcohol dependence	rs3131513	CLIC4, RUNX3	2 × 10^−6^	23942779
Autism spectrum disorder	rs9393484	SPTLC1P2	1 × 10^−6^	28540026
Bipolar disorder	rs9665626	SUFU, TRIM8	2 × 10^−9^	33479212
Depression	rs13270070	CLDN23, MFHAS1	8 × 10^−16^	33859377
Educational attainment	rs7606483	ALMS1P1, RNU6-111P, RPSAP28	3 × 10^−11^	35361970
General cognitive ability	rs7606483	RNU6-111P RPSAP28	2 × 10^−8^	29844566
Neuroticism	rs13270070	MFHAS1	6 × 10^−30^	27067015, 29255261, 29255261

**Table 4:** MAGMA Gene-Set Analysis. The table shows the top ten significant results from the gene-set enrichment analysis on the Gene Ontology (GO) terms from the Molecular Signature Database. Terms are listed in ascending order of significance. Additional information on the significant gene sets is reported in Supplementary Table S4. N. genes = number of genes; β = beta value; SE = standard error; Pbon = Bonferroni-corrected *p*-value.

Gene Set	N. Genes	β	SE	*p*-Value	Pbon
REACTOME_UREA_CYCLE	6	1.9558	0.30306	5.6363 × 10^−11^	9.5625 × 10^−7^
ROZANOV_MMP14_TARGETS_DN	26	0.8407	0.1762	9.2779 × 10^−7^	1.5739 × 10^−2^
ZHAN_MULTIPLE_MYELOMA_MS_DN	37	0.6046	0.1401	8.0401 × 10^−6^	1.1363 × 10^−1^
GOLDRATH_HOMEOSTATIC_PROLIFERATION	101	0.3758	0.0899	1.4548 × 10^−5^	2.4678 × 10^−1^
GOBP_REGULATION_OF_DNA_DEMETHYLATION	10	1.0509	0.2629	3.2099 × 10^−5^	5.4446 × 10^−1^
GOBP_POSITIVE_REGULATION_OF_DNA_DEMETHYLATION	8	1.1283	0.2841	3.5860 × 10^−5^	6.0822 × 10^−1^
GOBP_POSITIVE_REGULATION_OF_INTRINSIC_APOPTOTIC_SIGNALING_PATHWAY	39	0.5700	0.1469	5.2322 × 10^−5^	8.8738 × 10^−1^
GOBP_POSITIVE_REGULATION_OF_RELEASE_OF_SEQUESTERED_CALCIUM_ION_INTO_CYTOSOL	32	0.5695	0.1482	6.9526 × 10^−5^	1
GOBP_NEGATIVE_REGULATION_OF_NUCLEOCYTOPLASMIC_TRANSPORT	23	0.6782	0.1775	6.6904 × 10^−5^	1
GOBP_POSITIVE_REGULATION_OF_POST_TRANSCRIPTIONAL_GENE_SILENCING	13	1.0190	0.2674	6.0945 × 10^−5^	1

## Data Availability

The data supporting the findings are not publicly available, but they can be provided by the corresponding author (S.T.) upon reasonable request.

## Supplementary Materials

The following supporting information can be downloaded at: https://www.mdpi.com/article/10.3390/genes16040439/s1, Table S1: All SNPs in Linkage Disequilibrium (LD) with any of independent significant SNPs, Table S2: Genome-wide association studies on non-affective psychosis: suggestive and genome-wide significant signals, Table S3: List of SNPs reported in the GWAS Catalog resulting from the genome-wide association study (GWAS) of non-affective psychosis, Table S4: Significant gene-set and genes from MAGMA gene-sets enrichment analysis.
